# A Phytopharmacological Review on a Medicinal Plant: *Juniperus communis*

**DOI:** 10.1155/2014/634723

**Published:** 2014-11-11

**Authors:** Souravh Bais, Naresh Singh Gill, Nitan Rana, Shandeep Shandil

**Affiliations:** ^1^Department of Pharmacology, Rayat Institute of Pharmacy, Vpo Railmajra, Nawanshahr District, Punjab 144533, India; ^2^Rayat Institute of Pharmacy, Vpo Railmajra, Nawanshahr District, Punjab 144533, India

## Abstract

*Juniperus communis* is a shrub or small evergreen tree, native to Europe, South Asia, and North America, and belongs to family Cupressaceae. It has been widely used as herbal medicine from ancient time. Traditionally the plant is being potentially used as antidiarrhoeal, anti-inflammatory, astringent, and antiseptic and in the treatment of various abdominal disorders. The main chemical constituents, which were reported in *J. communis* L. are *α*-pinene, *β*-pinene, apigenin, sabinene, *β*-sitosterol, campesterol, limonene, cupressuflavone, and many others. This review includes the last 20 years journals and various books update on this plant, representing its pharmacological activity and health benefits against various diseases.

## 1. Introduction

**Figure 1:**
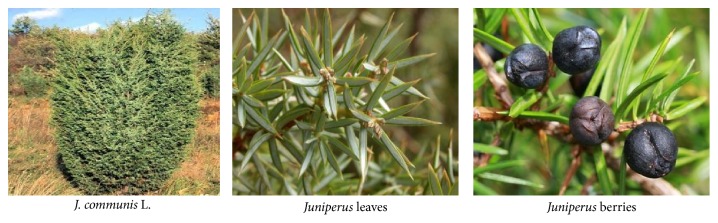
Images of* J. communis *L. plant.

Plants have been used as primary sources of disease treatments from ancient times and till to date a number of species have been reported to possess various pharmacological activities [1–3]. From ancient time herbs had been used by all cultures of the world including India that has one of the oldest, richest, and most diverse culture [4]. Advances in clinical research and quality control showed a greater value of herbal medicine in the treatment and overcome from many diseases [5].* Juniperus *genus is a well-known source of cedarwood oil which is widely distributed in the North hemisphere and it is used in folk medicine [6, 7].* J. communis *L. (Figure 1) is a shrub or small evergreen tree belonging to family Cupressaceae. The plant has been reported as diuretic, having anti-inflammatory properties [8, 9], antifungal activity [10], analgesic activity [11], hepatoprotective activity [12], antidiabetic and antihyperlipidemic activity [13], antimicrobial activity [14], antioxidant activity [15], antihypercholesterolemic activity [16], antibacterial activity [17], anticataleptic activity, and neuroprotective activity in Parkinson's disease [2, 18]. The analysis of the volatile fraction of* J. communis *berries was done by HS-SPME coupled to GC/MS for gin aromatization and more than 20 constituents have been reported [19].

## 2. Synonyms

- Sanskrit: Havusa, Matsyagandha
- Assamese: Arar, Abahal, Habbul
- Bengali: Hayusha
- Eng: Juniper Berry, Common Juniper
- Gujrati: Palash
- Hindi: Havuber, Havubair
- Kannada: Padma Beeja
- Marathi: Hosh
- Punjabi: Havulber
- Telugu: Hapusha
- Urdu: Abhal, Aarar.

## 3. Scientific Classification

- Species:* Juniperus communis*
- Class: Pinopsida
- Division: Pinophyta
- Order: Pinales
- Family: Cupressaceae
- Genus:* Juniperus*
- Binomial name:* J. communis* L.

## 4. Distribution

*J. communis* is found in Himachal Pradesh at an altitude of 3000 m–4200 m. It is mainly distributed in Manimahesh in Chamba, Kullu, Churdhar in Sirmour, Chhota and Bara Bhnghal in Kangra, and Kinnaur and Pattan valley in Lahaul-Spiti districts. The plant also grows in Europe south-western Asia, and North America [20].

## 5. Description 

### 5.1. Macroscopic

Fruit subspherical, purplish-black showing a “bloom” (0.5–1.0 cm in diameter): at the base are six, small, pointed, bracts arranged in 2 whorls, occasionally 3 or 4 whorls present; apex shows triradiate mark and depression indicating the suture; three hard, triangular seeds are embedded in the fleshy mesocarp, having terebinthne odour and bitter taste.

### 5.2. Microscopic

Seed coat shows 2-3 layers of thin-walled cells which are externally covered by a thin cuticle and which are internally followed by thick-walled polygonal sclerenchymatous cells. Endosperm and embryo are not distinct. Outer layer of fruit shows 3-4 large cubic or tabular cells having thick, brown porous walls. Sarcocarp consists of large, thin-walled, elliptical, loosely coherent cells, containing prismatic crystals of calcium oxalate and drops of essential oil [21].

## 6. Traditional Uses

See Table 1.

## 7. Phytochemical Screening

Dried powder of* J. communis *stems (200 g) was successively extracted with petroleum ether chloroform and ethanol (soxhlet). The marc was obtained which was successively air dried. Water extract was successively obtained by boiling with distilled water (2 h). Than it was filtered, concentrated, and dried in an oven. After that all the extracts were dissolved in their relevant solvents and were screened for phytoconstituents [22] (Table 2).

## 8. Chemical Constituents

It contains various chemical constituents including flavonoids, volatile oil, and coumarins.

### 8.1. Flavonoids

#### 8.1.1. Berries

**Figure 2:**
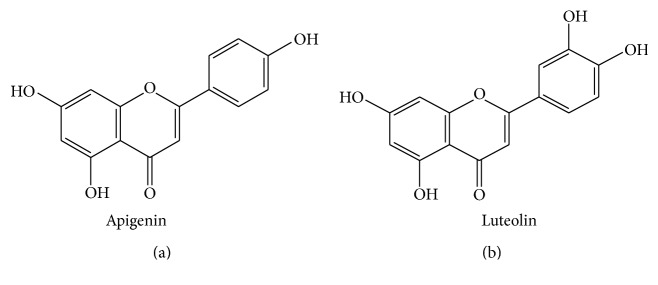


They contain apigenin, rutin, luteolin, quercetin-3-O-arabinosyl-glucoside, quercetin-3-o-rhamnoside quercitrin, scutellarein, nepetin, amentoflavone, and bilobetin [3, 23–27] (Figure 2).

#### 8.1.2. Leaves

They contain the cupressuflavone, hinokiflavone, biflavones, isocryptomerin amentoflavone, and sciadopitysin. The seeds contain haemagglutinin. Plant also contains several labdane diterpenes and diterpenoids (methanolic extract) [28].

### 8.2. Volatile Oil

**Figure 3:**
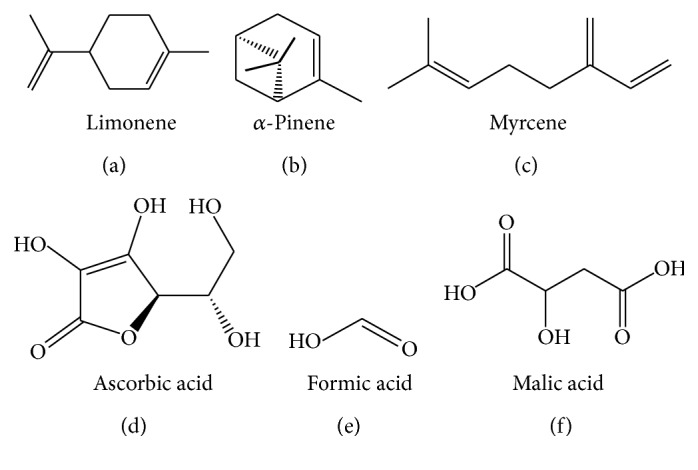


The juniper berry oil is largely comprised of monoterpene hydrocarbons such as *β*-pinene (5.0%), *α*-pinene (51.4%), sabinene (5.8%), myrcene (8.3%), and limonene (5.1%) [15] (Figure 3). The seeds and fruits of the plant contain d-*α*-pinene, camphene, pectins, glycolic acid, malic acid, formic acid, acetic acid, cyclohexitol, terpene, proteins, fermentable sugars, wax, gum, ascorbic acid, dihydrojunene, *β*-pinene, hydrocarbon-junene, cadinene, juniper, and camphor [29].

### 8.3. Coumarins

**Figure 4:**
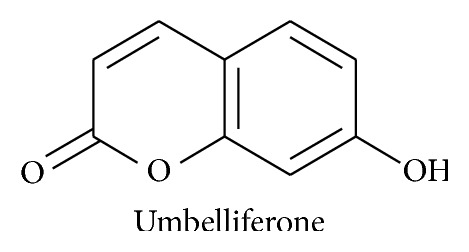


They contain umbelliferone; see Figure 4 [23].

### 8.4. Bicyclic Diterpenes

They contain imbricatolic acid, Junicedral,* trans*-Communic acid, diterpenes, isocupressic acid, aryltetralin, and lignan deoxypodophyllotoxin [6, 29]. Three new diterpene acids have been identified as 15-dien-18-oic acid, 7-oxo-13-epi-pimara-8, 7*α*-hydroxysandaracopimaric acid [30–32].

## 9. Pharmacological Activities

### 9.1. Hepatoprotective Activity

The hepatoprotective activity of* J. communis* in rats was determined by given CCl_4_ administration for 9 days. In CCl_4_ treatment group was showed significant increase in serum glutamic oxaloacetic transaminase (SGOT), serum glutamic pyruvic transaminase (SGPT), total bilirubin (TB), and alkaline phosphatase (ALP) values when compared to control group. There was significant decrease in the level of SGPT, SGOT, TB, and ALP in silymarin treated group. The abnormal high level of SGOT, SGPT, ALP, and bilirubin observed was due to CCl_4_ induced hepatotoxicity.* J. communis *reduced the increased levels of serum SGPT, SGOT, ALP, and bilirubin, which showed protection against hepatic cells (ethanol and aqueous extract show better protection) [12].

### 9.2. Anti-Inflammatory Activity

Anti-inflammatory activity of* J. communis* fruit has determined using isolated cells and enzymatic test. The plant showed varying degree of activity at 0.2 mg/mL in prostaglandin test and 0.25 mg/mL in platelet activating factor (PAF) test (aqueous extract).* J. communis *showed 55% prostaglandin inhibition and 78% PAF-exocytosis inhibition. The PAF activity was measured by inducing exocytosis of elastase. All plant extracts were studied on thin layer chromatography eluted with ethyl acetate/methanol/water [9].

### 9.3. Antioxidant Activity

Antioxidant activity has reported the* in vitro* antioxidant activity of plant using different assays like DPPH scavenging, superoxide scavenging, ABTS radical cation scavenging, and hydroxyl radical scavenging. The antioxidant effects of the oil were confirmed by* in vivo* study and created the possibility of blocking the oxidation processes in yeast cells by increasing the activity of the antioxidant enzymes [15].

### 9.4. Antidiabetic and Antihyperlipidemic Activity

*J. communis *was reported to have antidiabetic and antihyperlipidemias activity in streptozotocin- (STZ-) nicotinamide induced diabetic rats.* J. communis *(methanolic extract, 100 mg/kg and 200 mg/kg p.o.) was administered except to the group that received (glibenclamide 10 mg/kg). Biochemical estimation and fasting blood glucose levels were estimated on the 21st day. The methanolic extract of* J. communis *mediated significant (*P* < 0.01) reduction in blood glucose levels and increase in HDL levels in diabetic rats. Glibenclamide (standard drug) showed a significant decrease in the level of SGPT and SGOT. Methanolic extract of* J. communis *showed a significant anti diabetic and antihyperlipidemic activity [13].

### 9.5. Analgesic Activity

Banerjee and collaborators [11] reported the analgesic activity of* J. communis* using methanolic extract. The methanolic extract was given at a dose of 100 mg/kg and 200 mg/kg and evaluated for its analgesic activity. Acetylsalicylic acid was used as standard (100 mg/kg).* In vivo* the extract was evaluated by different tests like formalin test, acetic acid induced writhing, and tail flick tests.* J. communis *showed a significant (*P* < 0.01) and dose dependent effect on inhibition of writhing response and dose dependent inhibition in the late phase as compared to aspirin (*P* < 0.01), formalin test. The blocking effect of naloxone (2 mg/kg i.p.) confirms the central analgesic activity. The plant showed significant antinociceptive activity and it has been established that the methanolic extract of* J. communis *acts both peripherally and centrally [11].

### 9.6. Antibacterial Activity

The leaf extracts (methanol, ethanol, chloroform, and hexane aqueous) of* J. communis *were evaluatedagainst five pathogenic multidrug resistant bacteria (*Erwinia chrysanthemi, Escherichia coli, Bacillus subtilis*,* Agrobacterium tumefaciens, *and* Xanthomonas phaseoli*), by using disc diffusionmethod. It has been estimated that all extracts of leaves of* J. communis *were effective against the pathogenic bacteria except aqueous extract. The hexane extract showed more activity as compared to other extracts (hexane > ethanol > methanol > chloroform extract). The methanolic extract of* J. communis *was found to be very effective as compared to standard antibiotics (ampicillin 10 mcg and erythromycin 15 mcg) [17].

### 9.7. Antimicrobial Activity

The berries of* J. communis* were reported to have antimicrobial activity and volatile oils were analyzed by GC-FID and GC-MS. Its oil was investigated for its antimicrobial activity and the activity was tested against* Escherichia coli, Staphylococcus aureus*,* Hafnia alvei, *and* Pseudomonas aeruginosa*. DMF solution with three different concentrations of essential oil (1, 3, and 5 mg/mL) was prepared which were applied on disc for the measurement of the diameter of the zone of inhibition around the disc. The chromatographic analysis of the essential oil of* J. communis *allowed identifying 41 components which represent 96% of the oil total composition (Table 3). The main chemical constituents in* J. communis *were *α*-cadinol (1.6%), *α*-pinene (36.2%), *β*-myrcene (21.1%), *α*-humulene (1.5%), epi-*α*-bisabolol (1.3%), germacrene D (2.2%), spathulenol (1.4%), and germacrene B (1.1%). The present study shows the chemical composition of* J. communis *from east part of Kosova.* J. communis* was active against* Escherichia coli, Staphylococcus aureus*, and* Hafnia alvei *except* Pseudomonas aeruginosa *which is resistant* to J. communis *[14].

### 9.8. Antifungal Activity

The aerial* parts of J. communis *were isolated by hydrodistillation for their essential oil with 0.1 and 0.3% yield. The oils were then tested for their antifungal (*in vitro*) activity against two fungi,* Rhizoctonia solani *and* Rhizopus stolonifer*. The essential oils obtained from* J. communis *showed antifungal activity against both fungi:* J. communis *(EC50: 0.554 and 0.704 mg/mL). The antifungal activity of* J. communis *is mainly due to the presence of high content of oxygenated monoterpenes [8].

### 9.9. Antimalarial Activity

The leaves and twigs (stems) of eight plants were isolated for their essential oil by hydrodistillation method (*Juniperus communis, Artemisia vulgaris, Myrtus communis, Lavandula angusti/olia, Eucalyptus globulus, Rosmarinus officinalis, Origanum vulgare, *and* Salvia officinalis*) and were analyzed by GC-FID and GC-MS. The essential oil obtained from these plants was then tested for their antimalarial activity on* Plasmodium falciparum*. There were two strains of* Plasmodium falciparum*: FcBl Columbia and a Nigerian chloroquine-sensitive strain. Two concentrations ranged from 150 *μ*g/mL to 1 mg/mL showed 50% inhibition of the growth of the parasite (*in vitro*) and the effect was obtained after 24 and 72 h.* Myrtus communis* and* Rosmarinus officinalis* oils at a concentration ranged from 150 to 270 *μ*g/mL showed best result against* Plasmodium falciparum* [33].

### 9.10. Antihypercholesterolemic Activity

*J. communis *fruit oil has been evaluated for its antihypercholesterolemic activity. The biochemical parameters and the histopathologic effects on kidney tissue were evaluated. Healthy Wistar albino rats of 200–250 gm in weight were used for this study. The rats were divided into 5 groups; first group is control group in which the animal was fed with normal pellet chow. The second group is cholesterol group which was fed with pellet chow containing 2% of cholesterol, and the third group is* J. communis *(JCL) group which was further divided into three subgroups 50 JCL, 100 JCL, and 200 JCL groups which were fed with 50, 100, and 200 mg/kg* J. communis *oil, with addition to the 2% cholesterol-containing pellet chow. JCL was administered by a gavage needle (dissolved in 0.5% sodium carboxy methyl cellulose (SCMC)). After 30 days blood and kidney tissue samples were taken and biochemical estimation and histopathological investigation were done. The 200 mg/kg JCL group showed a significant increase in blood urea nitrogen (BUN) and creatinine levels. The cholesterol group showed a significant increase in Ox-LDL levels. When the cholesterol was given along with 200 mg/kg* J. communis *then there was no significant increase in the level of Ox-LDL. So the study showed its antihypercholesterolemic effect [16].

### 9.11. Anticataleptic Activity

Anticataleptic study was carried out to evaluate the effects of methanolic extract of* J. communis *(MEJC) leaf in reserpine induced catalepsy in rats. Catalepsy was induced by intraperitoneal (i.p.) administration of reserpine (2.5 mg/kg, i.p.). The methanolic extract at 100 and 200 mg/kg (i.p.) was screened for its efficacy against reserpine induced catalepsy in rats. The MEJC extract was found to reduce catalepsy significantly (*P* < 0.001) as compared to the reserpine treated rats; maximum reduction was observed at a dose of 200 mg/kg [18].

### 9.12. Neuroprotective Activity

Neuroprotective activity of* J. communis* (MEJC) was evaluated in chlorpromazine (CPZ) induced Parkinson's model in rats. The two doses (100 and 200 mg/kg, i.p.) have been selected on the basis of lethal dose (LD_50_) in mice. The plant was evaluated for various behavior parameters like catalepsy (bar test), muscle rigidity (rot rod test), and locomotor activity (actophotometer) and its effect on biochemical parameters (TBARS, GSH, nitrite, and total protein) in rats brain.* J. communis* showed a significant (*P* < 0.001) neuroprotective effect of MEJC against CPZ induced Parkinson's like symptoms or anti-Parkinson's activity [2].

## 10. Conclusion

The extensive literature survey revealed that* J. communis *L. is an important medicinal plant due to its traditional uses to treat diseases and presence of many active chemical constituents which are responsible for various medicinal and pharmacological properties. Further evaluation needs to be carried out on* J. communis *L. in order to confirm its medicinal uses and development of formulations containing this plant for their practical clinical applications, which can be used for the welfare of mankind.

## Figures and Tables

**Table 1:** Traditional uses of *J. communis* L. plant.

Part	Traditional use	Reference
Berries	Carminative, urinary antiseptic, diuretic, emmenagogue, sudorific, digestive, and anti-inflammatory.	[7, 13]
Aerial parts	Used for acute and chronic cystitis, albuminuria, catarrh of the bladder, renal suppression, leucorrhoea, and amenorrhoea.	
Fruit	Used as antiseptic, stimulant, disinfectant, styptic, chronic Bright's disease, migraine, dropsy, rheumatic and painful swellings, piles, and infantile tuberculosis.	[7, 14]
Bark	Nephrotic dropsy of children, asthma, gonorrhoea, pulmonary blennorrhoea, arthritis, respiratory affections, diabetes, bladder affections, chronic pyelonephritis, cough, abdominal disorders, and skin affections.	

**Table 2:** Phytochemical screening of *J. communis* [12].

Serial number	Phytoconstituents	Petroleum ether extracts	Chloroform extracts	Methanol extracts	Aqueous extracts
1	Alkaloids	−	*+ *	*+ *	*+ *
2	Flavonoids	−	*+ *	*+ *	*+ *
3	Glycosides	−	−	*+ *	*+ *
4	Tannins and phenolic compounds	−	−	*+ *	*+ *
5	Steroids/triterpenoides	*+/*−	*+/+ *	*+/+ *	*+/+ *
6	Carbohydrates	−	−	−	−
7	Proteins and amino acids	−	−	−	−

**Table 3:** Essential oil components of *J. communis* L. [8].

	Rt	%

Monoterpene hydrocarbons		
(i) *α*-Pinene	4.40	1.95
(ii) dl-Limonene	6.33	0.96
(iii) *α*-Pinene	10.78	0.80
(iv) (+)-4-Carene	12.44	3.86
(v) Bicyclo[4.1.0]hept-2-ene,3,7,7-trimethyl	12.81	0.71

	Rt	%

Sesquiterpene hydrocarbons		
(i) *α*-Cedrene	12.98	0.15
(ii) *α*-Cadina-4,9-diene	13.08	0.93
(iii) Cedrene	13.64	4.04
(iv) Gamma. 1-cadinene	14.09	1.00

	Rt	%

Oxygenated monoterpenes		
(i) 1-Indanone	7.60	1.15
(ii) Linalool	7.85	2.34
(iii) 2,3,3-Trimethyl-3-cyclopentene acetaldehyde	8.35	2.09
(iv) 5-Decene-1-ol	10.89	2.60

	Rt	%

Oxygenated sesquiterpenes		
(i) Cedrene epoxide	18.94	2.79
